# Renal carcinoma CD105−/CD44− cells display stem-like properties *in vitro* and form aggressive tumors *in vivo*

**DOI:** 10.1038/s41598-020-62205-6

**Published:** 2020-03-25

**Authors:** M. Fiedorowicz, M. I. Khan, D. Strzemecki, J. Orzeł, M. Wełniak-Kamińska, A Sobiborowicz, M. Wieteska, Z. Rogulski, L. Cheda, W. Wargocka-Matuszewska, K. Kilian, C. Szczylik, A. M. Czarnecka

**Affiliations:** 1 Mossakowski Medical Research Centre, Polish Academy of Sciences, Warsaw, Poland; 2 Department of Oncology with Laboratory of Molecular Oncology, Military Institute of Medicine, Warsaw, Poland; 3 Present Address: Department of Otolaryngology - Head & Neck Surgery, Western University, London, ON N6A 3K7 Canada; 4 Faculty of Electronics and Information Technology, Warsaw University of Technology, Warsaw, Poland; 5 Faculty of Medicine, Medical University of Warsaw, Warsaw, Poland; 6 Department of Soft Tissue/Bone Sarcoma and Melanoma, Maria Sklodowska-Curie Memorial Cancer Center and Institute of Oncology, Warsaw, Poland; 7 Faculty of Chemistry, Biological and Chemical Research Centre, University of Warsaw, Warsaw, Poland; 8 Heavy Ion Laboratory, Faculty of Physics, University of Warsaw, Warsaw, Poland; 9 Department of Oncology, European Health Centre, Otwock, Poland; 10 Medical Center for Postgraduate Education, Warsaw, Poland; 11 Present Address: Department of Soft Tissue/Bone Sarcoma and Melanoma, Maria Sklodowska-Curie National Research Institute of Oncology, Warsaw, Poland

**Keywords:** Cancer imaging, Cancer stem cells, Urological cancer

## Abstract

Clear cell renal cell carcinoma (ccRCC) is the most common kidney cancer. Prognosis for ccRCC is generally poor since it is largely resistant to chemo- and radiotherapy. Many studies suggested that cancer stem cells/tumor initiating cells (CSCs/TICs) are responsible for development of tumor, disease progression, aggressiveness, metastasis and drug resistance. However, tumorigenic potential of CSCs/TICs isolated from established RCC cell lines – basic ccRCC research model – has never been investigated *in vivo*. CD105+, CD105−, CD44+ and CD44− as well as CD44−/CD105− CD44+/CD105+ and CD44−/CD105+ cells were isolated from Caki-1 RCC cell line, confirming coexistence of multiple subpopulations of stem-related phenotype in stable cell line. Sorted cells were injected subcutaneously into NOD SCID mice and tumor growth was monitored with MRI and PET/CT. Tumor growth was observed after implantation of CD105+, CD44+, CD44−, CD44−/CD105+ and CD44−/CD105− but not CD105− or CD44+/CD105+. Implantation of CD44−/CD105− cells induced tumors that were characterized by longer T1 and distinct metabolic pattern than other tumors. All the tumors were characterized by low uptake of [18F]FDG. CD105+ and CD44− tumors expresses Nanog and Oct-4, while CD44− tumors additionally expressed endothelial cell marker - CD31.

## Introduction

Renal cell carcinoma (RCC), is the 10th malignancy worldwide and the most frequent type of kidney cancer in adults. Each year in Europe approximately 88 400 patients are diagnosed with RCC; the incidence and mortality of RCC are rising at a rate of 2–3% per decade, therefore novel therapies directed against RCC are needed. At the same time despite advancements in diagnostic techniques, up to 30% of newly diagnosed patients already present with metastases, and a large portion of patients that undergo surgical treatment experience the RCC recurrence, therefore drugs targeted against metastasis initiating cells would be of great interest in the future^1,2^. Cancer stem cells (CSCs) are characterized by the potential to self-renew, high tumorigenicity in nude mice and the ability to efficiently reconstitute all tumor subpopulations and primary tumor phenotype^3–5^. CSCs are also responsible not only for cancer development, but also for disease recurrence, progression and metastatic spread, together with cancer aggressiveness, including treatment resistance such as chemo/radiotherapy, and targeted treatment^6,7^, therefore basic research with careful model selection to understand their biology is mandatory to define novel potential therapeutic targets for all RCC subtypes^8,9^.

Presence of cancer stem cells (also called tumor/metastasis initiating cells) or/and cancer progenitor cells presence was over last years demonstrated in renal cancer cell lines by us and other research groups^10–12^. As biomarkers of CSCs multiple surface proteins have been indicated including: CD105^13,14^, CD133^15,16^, CD44^10^, or CXCR4^17^; but the co-expression of multiple membrane markers on RCC-CSCs is not defined^18,19^. Moreover *in vivo* growth characteristics of different RCC cells subpopulations (i.e. CD105+ vs. CD44+) has neither been described nor directly compared until now, therefore the significance of specific markers in the isolation of RCC-CSCs has not been elucidated. Until today no comprehensive reports on RCC-CSC derived tumors imaging *in vivo* were published. Most widely recognized RCC-CSCs biomarker - endoglin that is CD105 - surface expression is to be distinctive for these cells, and within the tumor only a small subpopulation is expected to express this protein, as CSCs usually represent minor fraction of the total tumor mass. Moreover, our previous *in vitro* work shown that CD105 expression is cell-line specific, transient or time-variable, and oxygen-tension, growth conditions and growth factors supplementation dependent^12,20,21^. Additionally, our analysis revealed that CD105+ subpopulation of cells isolated from - metastatic papillary VHL wt - RCC ACHN cell line also express CD44, CD73, CD90, CD146 and alkaline phosphatase (AP)^12^. The others have shown that spheres derived from HEK293T, ACHN, Caki‐1, and 786O renal cancer cell lines as well as CD105+ cells isolated from RCC specimens showed the presence of a CD44+ population with self‐renewal properties, sphere formation capability and resistance to therapy^22^. These results have convinced us that on-time analysis expression of multiple markers is indispensable for reliable characterization of RCC-CSCs, as we have primarily shown for ACHN and Caki-1 cell lines^12^. This study was designed to verify *in vivo* tumor formation potential of these preselected populations of ccRCC cells^9,12^ and therefore identify potential tumor initiating cells - referred as cancer stem cells in an animal model. We also aimed to describe their growth characteristics *in vitro* and *in vivo*.

## Results

### CD105/CD133/CD44/CXCR4 subpopulations are found in RCC cell lines

**Figure 1:**
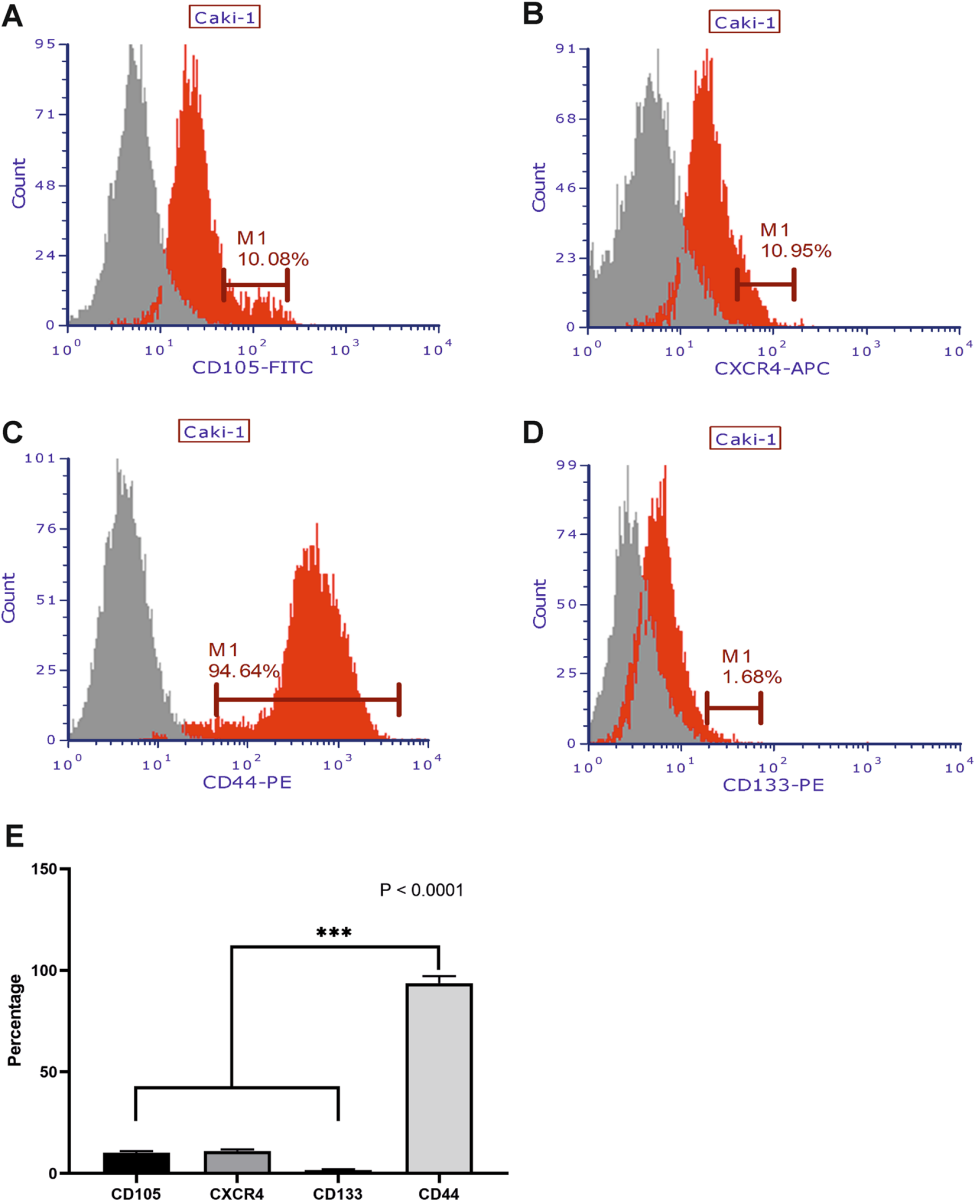
Flow cytometric analysis of CSCs subpopulations in Caki-1 cell line. Red histograms showing percentage of **(A)** CD105−CSCs **(B)** CXCR4-CSCs **(C)** CD44−CSCs and **(D)** CD133-CSCs observed in Caki-1 cells. **(E)** Bar-graph showing quantification of different CSCs subpopulations. Significant difference was observed between CD44 and other CSCs markers (CD105, CXCR4 and CD133). Red histogram represents stained cells, while grey histogram represents unstained cells. ***P < 0.001.

**Figure 2:**
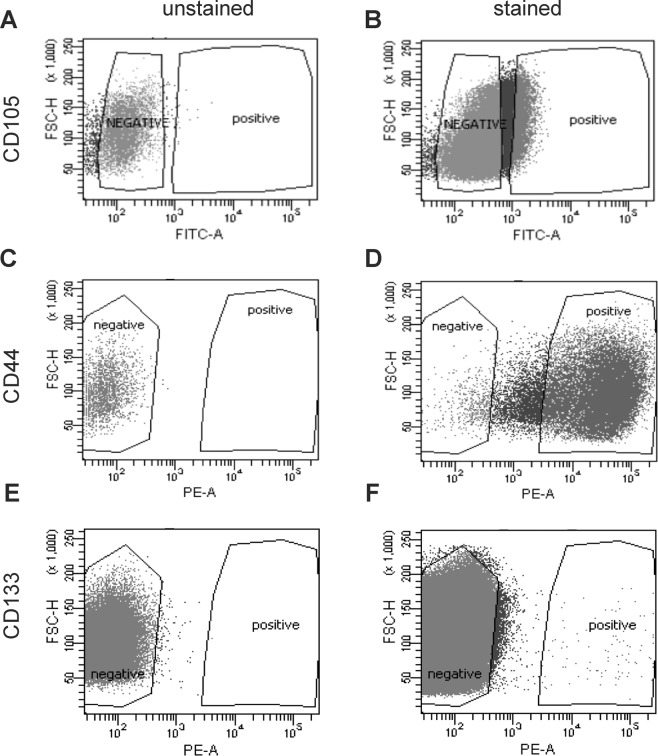
Sorting of CSCs based on FACS analysis by FACS Aria II cell sorter. Dot plots **(A) (C)** and **(E)** showing unstained cells before FACS sorting and dot plots showing stained cells positive for **(B)** CD105−CSCs **(D)** CD44−CSCs and **(F)** CD133-CSCs from Caki-1 cell line. These CSCs were sorted and culture in normoxic condition in FreeStyle™ 293 medium before xenograft experiments. The percentage of sorted cells positive for CD105, CD44, and CD133 makers were approximately 9%, 96% and 1%, respectively.

We have confirmed the presence of different stem-like subpopulations in clear cell RCC cell line Caki-1. This cell line expressed four potential stem cell marker subpopulations (CD105, CD133, CD44 and CXCR4) as found in our flow cytometric analysis **(**Fig. 1**)**. The percentages of CD105, CD133, CD44, and CXCR4 positive cells were variable in Caki-1 cells. CD105+ and CXCR4+ cells subpopulations were found with similar percentage within the Caki-1 cells, i.e. 10.08% and 10.95%, respectively. **(**Fig. 1A,B**)**. In contrast almost all Caki-1 cells were found positive for CD44 expression (94.64%), while CD133+ cells represents very low fraction of the Caki-1 cells (1.68%) **(**Fig. 1C,D**)**. CD44+ subpopulation was significantly larger than other tested subpopulations and results were statistically significant (Fig. 1E). Based on these results we isolated CD105+, CD105−, CD44+, CD44−, CD133+ and CD133− cell subpopulations from Caki-1 cell line for downstream analyses **(**Fig. 2**)**.

### Tumor 3D spheres derived from Caki-1 cells expressed stem cell markers

**Figure 3:**
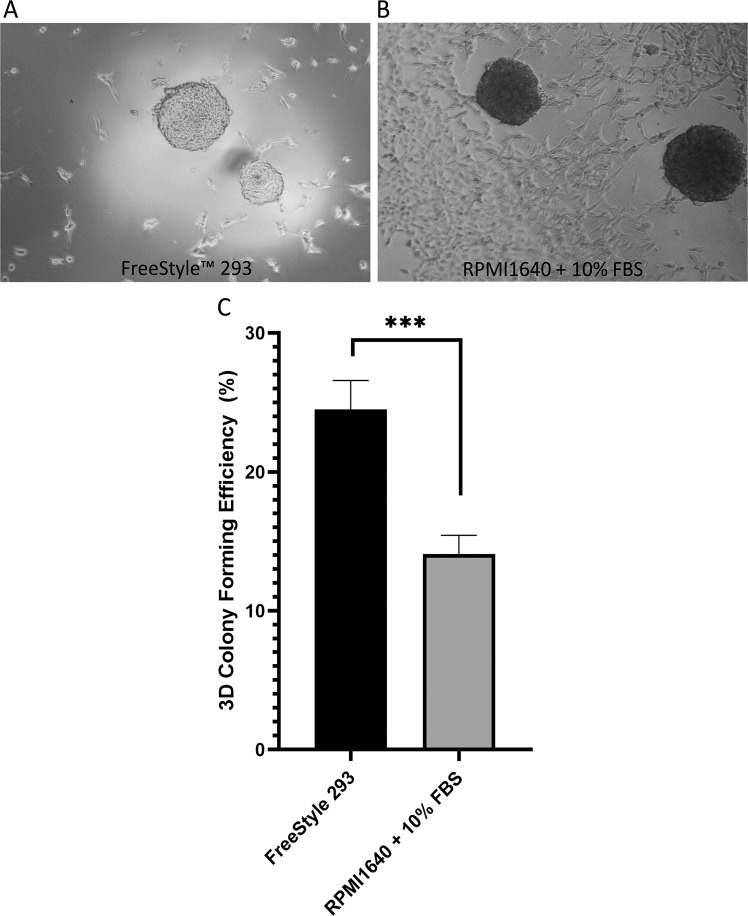
Generation of 3D spheres derived from unsorted Caki-1 cells in FreeStyle™ 293 and RPMI-1640+10%FBS medium. Representative pictures of 3D spheres formed by Caki-1 cells in **(A)** FreeStyle™ 293 and **(B)** RPMI-1640+10%FBS. Caki-1 cells cultured in low-attachment plate tend to attach more in RPMI-1640+10%FBS media in comparison to FreeStyle™ 293. **(C)** Quantitative analysis of 3D spheres formation in FreeStyle™ 293 and RPMI-1640+10%FBS medium. Scale bar = 100 μm and *** P< 0.001.

**Figure 4:**
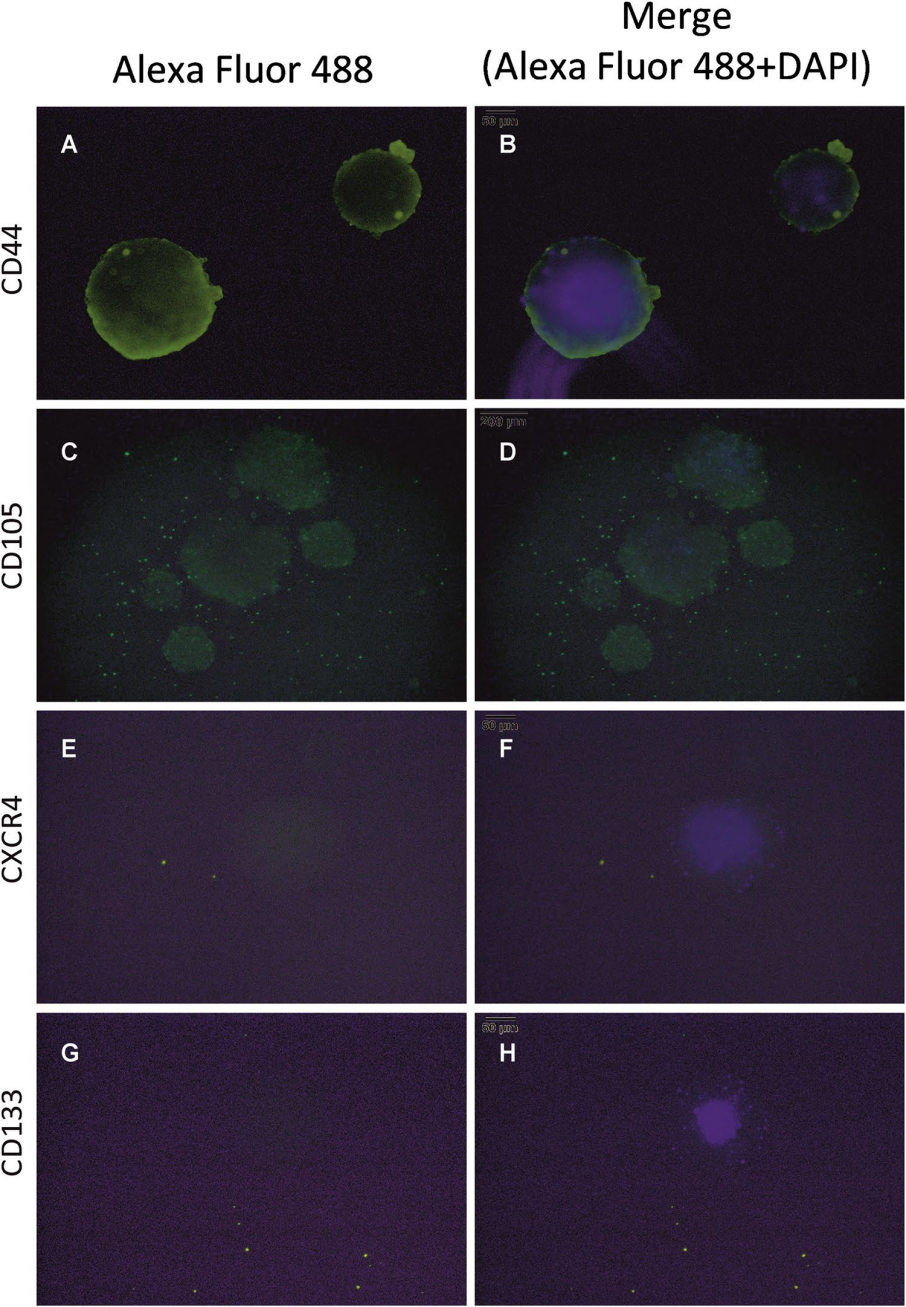
Staining of different CSCs-markers on 3D spheres derived from unsorted Caki1-cells. 3D spheres were grown in FreeStyle™ 293 and stained for CSCs-markers **(A)** CD44, **(C)** CD105, **(E)** CXCR4, and **(G)** CD133. 3D spheres were stained with primary antibody (e.g. CD44, CD105, CXCR4 and CD133) + secondary antibody Alexa Fluor 488 (green) and counter-stained with the nuclear dye DAPI (blue). Positive spheres were shown as green. Merged fluorescence of Alexa Fluor 488 and DAPI is shown in **(B), (D), (F), (H)**. Scale bar = 100 μm.

In our study, we have confirmed that Caki-1 cells form 3D tumor spheres **(**Fig. 3**)**. Spheres were enriched in cells expressing stem-like cell markers including membranous proteins CD105, CD133, CD44, and CXCR4. CD44 protein was highly expressed in the Caki-1 sphere-forming cells (Fig. 4A,B), while lower expression of CD105 and CXCR4 proteins was found in these spheres **(**Fig. 4C–F**)**. Cells growing in 3D spheres have very weak expression of CD133 protein **(**Fig. 4G,H**)**. Expression of stem-related markers in 3D culture (Fig. 4) corresponds with the findings of cell status in 2D growth (Figs. 1 and 2**)**, in which we have observed similar pattern of surface marker expressed by Caki-1 cells.

### Co-expression of multiple RCC-CSCs markers

**Figure 5:**
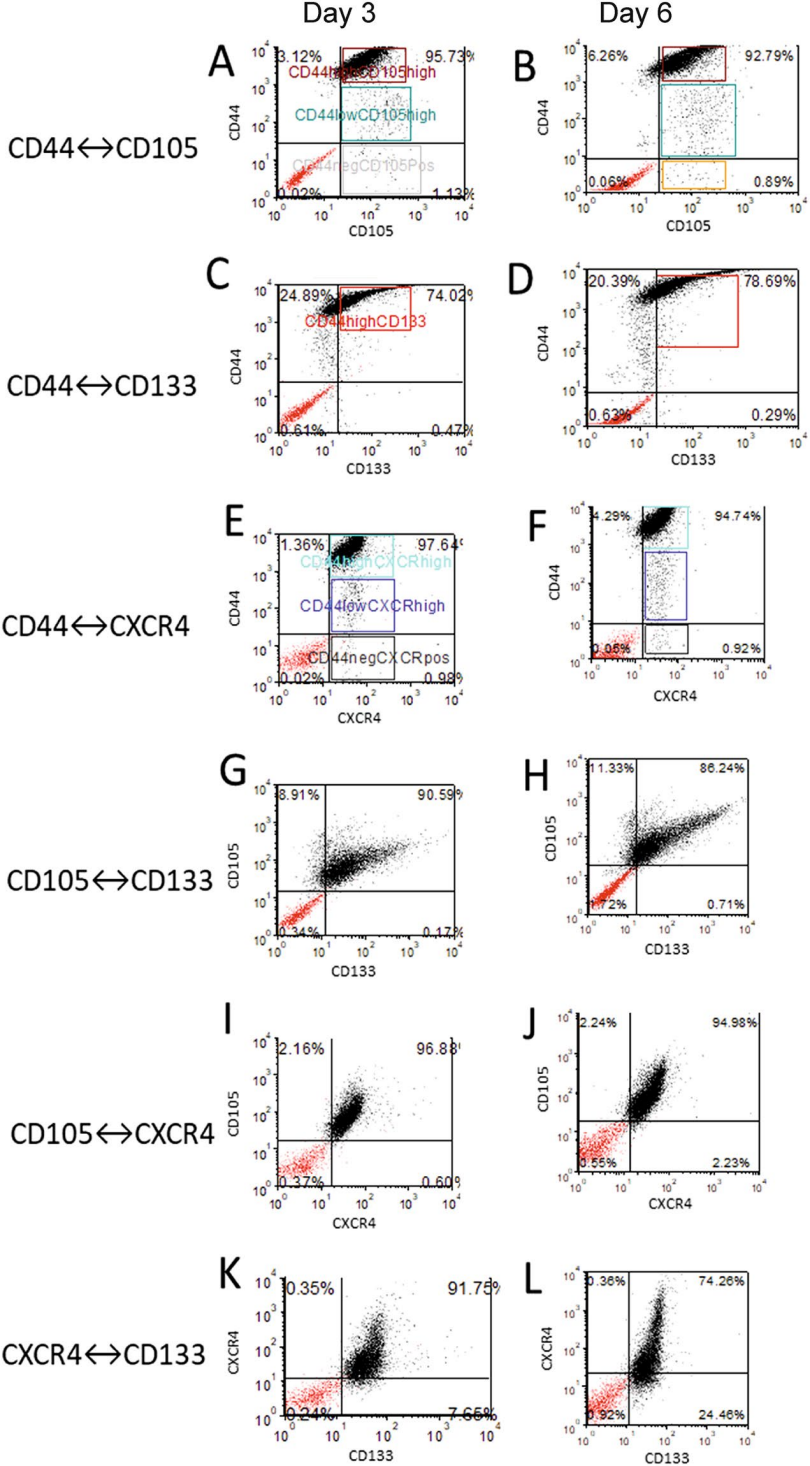
Co-expression analysis of multiple CSCs markers in Caki-1 cells. Dot plots (**A,B**) showing co-expression analysis of CD44+/CD105+ cells. Dot plots (**C**,**D**) showing co-expression analysis of CD44+/CD133+ cells. Dot plots (**E,F**) showing co-expression analysis of CD44+/CXCR4+ cells. Dot plots (**G,H**) showing co-expression analysis of CD105+/CD133+ cells. Dot plots (**I,J**) showing co-expression analysis of CD105+/CXCR4+ cells. Dot plots (**K**,**L**) showing co-expression analysis of CXCR4+/CD133+ cells. Red dot plots represent unstained cells and grey dots represent stained cells. Boxes between the dot plots represents high and low positive cells.

Expression of stem-cell markers changes with the different environment conditions like culturing cells for a longer period in a standard culture condition. We next wanted to investigate if co-expression of RCC-CSCs markers changes with the time analysis. We analyzed co-expression of RCC-CSCs markers after day 3 and day 6 to see if there is any difference in expression of these markers **(**Fig. 5**)**. In Caki-1 cells multiple co-expressed CSC subpopulations were found. The most common double positive subpopulation was CD44+/CD105+, this group was further subdivided into two subgroups as CD105 cells expressing high or low expression of CD44 **(**Fig. 5A,B**)**. Similar trend was found for co-expression of CD44+/CXCR4+ cells, with subpopulation of CXCR4+ and CD44 low and CD44 high expressing cells **(**Fig. 5E,F**)**. In contrast, in CD44+ cells CD133 was not expressed **(**Fig. 5C,D**)**. Of note, CD133+ subpopulation of Caki-1 cells did not co-express CD105 but expressed CXCR4 **(**Fig. 5G–L**)**. Interestingly, we found that CD105 and CXCR4 were co-expressed in a subpopulation of Caki-1 cells.

### Tumor growth

**Figure 6:**
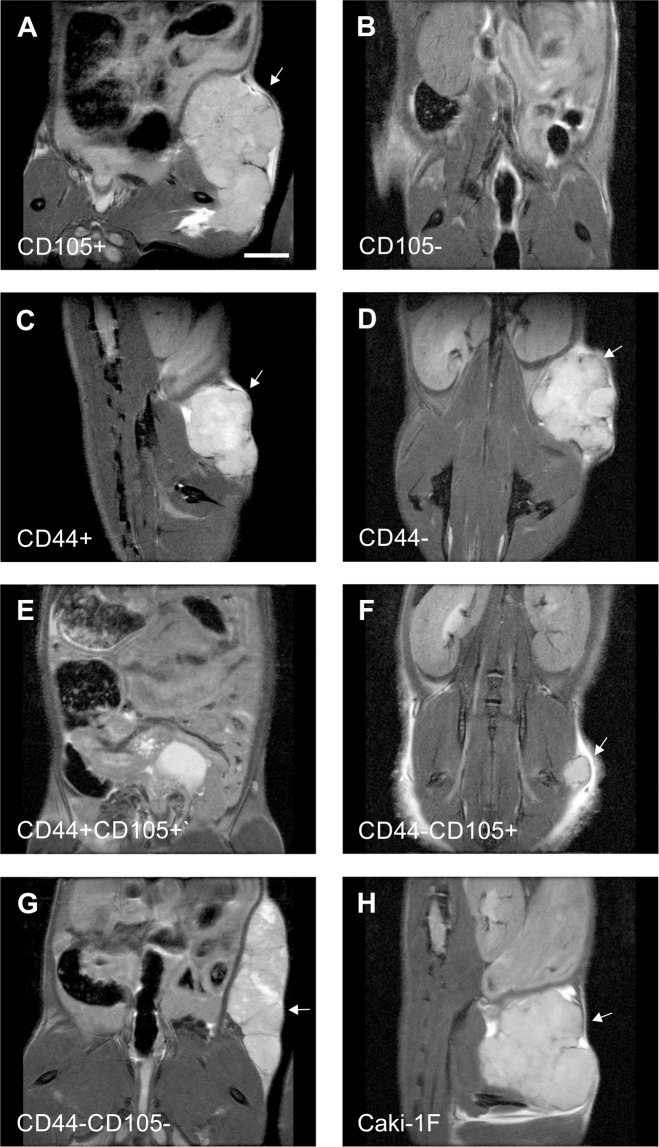
Anatomical T2-weighted MR images of the tumors that grew in NOD SCID mice 7 weeks after implantations of various subpopulations of Caki1F cells: CD105+ (**A**), CD105− (**B**), CD44+ (**C**), CD44− (**D**), CD44+/CD105+ (**E**), CD44−/CD105+ (**F**), CD44−/CD105− (**G**) or the unsorted Caki-1F cells (H). Arrows point the tumors. Scale bar represents 5 mm.

**Figure 7:**
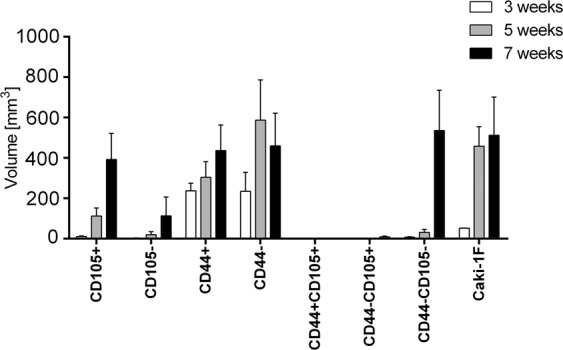
Volumes of the tumors that grew in NOD SCID mice after implantation of various subpopulations of Caki1F cells: CD105+, CD44+ (**B**), CD44− (**C**), CD44−/CD105+ (**D**), CD44−/CD105− (**E**) or the unsorted Caki-1F cells (**F**). Means ± SD.

Tumor growth after implantation of different subpopulation of Caki-1 cells was analyzed *in vivo* by T2-weighted magnetic resonance imaging (Fig. 6) and the resulting images were manually segmented to evaluate tumor volumes (Fig. 7). Small tumors were already observed 3 weeks after implantation of unsorted Caki-1 cells (52.0 ± 1.3 mm^3^), after 5 weeks the mean tumor volume was 457.8 ± 236.4 mm^3^ and 512.1 ± 423.8 mm^3^ after 7 weeks (Fig. 6H).

Tumor growth was also observed after implantation of CD105+ cells (392.2 ± 428.0 mm^3^ after 7 weeks, Fig. 6A) but no growth or very small tumors were observed after implantation of CD105− subpopulation (Fig. 6B). Similar growth rate was observed in CD44+ and CD44- subpopulations of Caki-1 cells (436.3 ± 127.1 vs. 459.9 ± 227.8 mm^3^ after 7 weeks, Fig. 6C,D).

However, no tumor growth was observed after implantation of CD44+/CD105+ cells (Fig. 6E) and small tumors were present after implantation of CD44−/CD105+ cells (8.8 ± 0.9 mm^3^ after 7 weeks, Fig. 6F). Implantation of CD44−/CD105− subpopulation of Caki-1 cells led to formation of specific tumors in all inoculated animals. The tumors were relatively small in the earlier timepoints (10.3 ± 5.0 mm^3^ at 3 weeks and 44.3 ± 31.3 mm^3^ at 5 weeks). However, 7 weeks after the implantation of CD44−/CD105− cells the tumors reached volume of 642.3 ± 413.4 mm^3^ (Fig. 6G).

### Angiography

**Figure 8:**
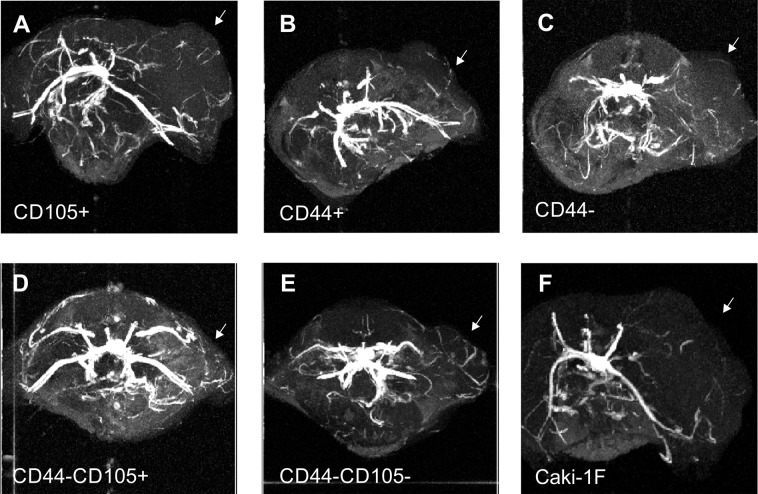
Representative MR angiography of the tumors that grew in NOD SCID mice 7 weeks after implantations of various subpopulations of Caki1F cells: CD105+ (**A**), CD44+ (**B**), CD44− (**C**), CD44−/CD105+ (**D**), CD44−/CD105− (**E**) or the unsorted Caki-1F cells (**F**). Arrows point the tumors.

MR *time of flight* angiography (i.e. without contrast agent) was used to track changes in vascularization in the course of tumor development (Supplementary Fig. 3). It revealed some vascularization in all the groups of animals that developed tumors at 7 weeks after the implantation of Caki-1 cells or their subpopulations (Fig. 8A–F). New tumor vessels were the most prominent in the CD105−/CD44− tumors (Fig. 8E).

### Relaxometry

**Figure 9:**
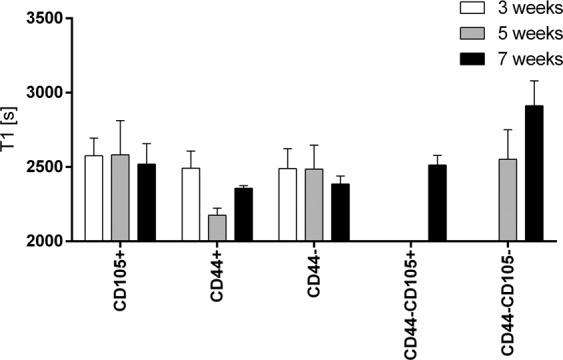
T1 relaxation time measured in ROIs centered on tumors that grew in NOD SCID mice after implantations of various subpopulations of Caki1F cells: CD105+, CD44+, CD44−, CD44−/CD105− or the unsorted Caki-1F cells. Means ± SD.

T1 relaxation times were measured *in vivo* for all the tumors that developed after implantation of the RCC cells subpopulations (Fig. 9). After 3 and 5 weeks the measured T1 and did not differ significantly between the groups. However, 7 weeks after the implantations we noted a significant increase in T1 relaxation time in C105−/CD44− group (2552 ± 199 vs 2912 ± 167, 5 weeks vs. 7 weeks, p < 0.05).

### Metabolic characteristics of the tumours

**Figure 10:**
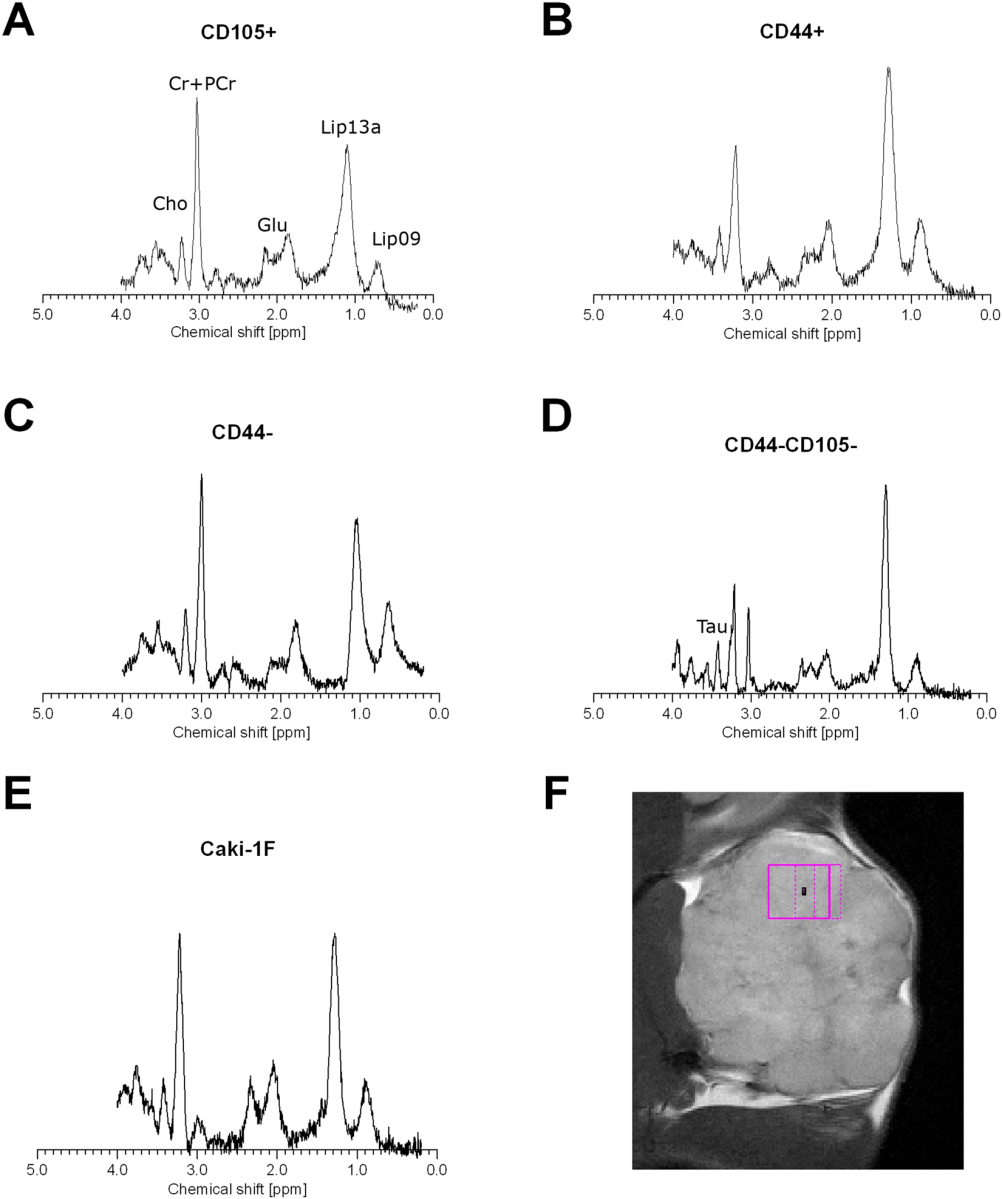
Representative MR spectra of the tumors that grew in NOD SCID mice 7 weeks after implantations of various subpopulations of Caki1F cells: CD105+ (**A**), CD44+ (**B**), CD44−, CD44−/CD105− (**D**) or the unsorted Caki-1F cells (**E**). The last panel (**F**) shows a representative localization of the volume of interest (VOI). Cho – choline, Cr+PCr – creatine + phosphocreatine (total creatine), Tau – taurine, Glu – glutamate, Lip09 – lipids 0.9 ppm, Lip13a – lipids 1.3 ppm.

Metabolic status of the tumors was analyzed with *in vivo* localized proton magnetic resonance spectroscopy. We have identified several lipid signals (mainly Lip13a and Lip09), choline compounds (Cho), creatine and phosphocreatine (Cr+PCr, tCr), glutamate (Glu) in all the tumors. Representative spectra are shown in Fig. 10. In CD105−/CD44− tumors there was much stronger lipid signal than in all other tumors. Moreover, we noted a presence of additional signal that was identified as taurine (Tau, Fig. 10D).

### PET

**Figure 11:**
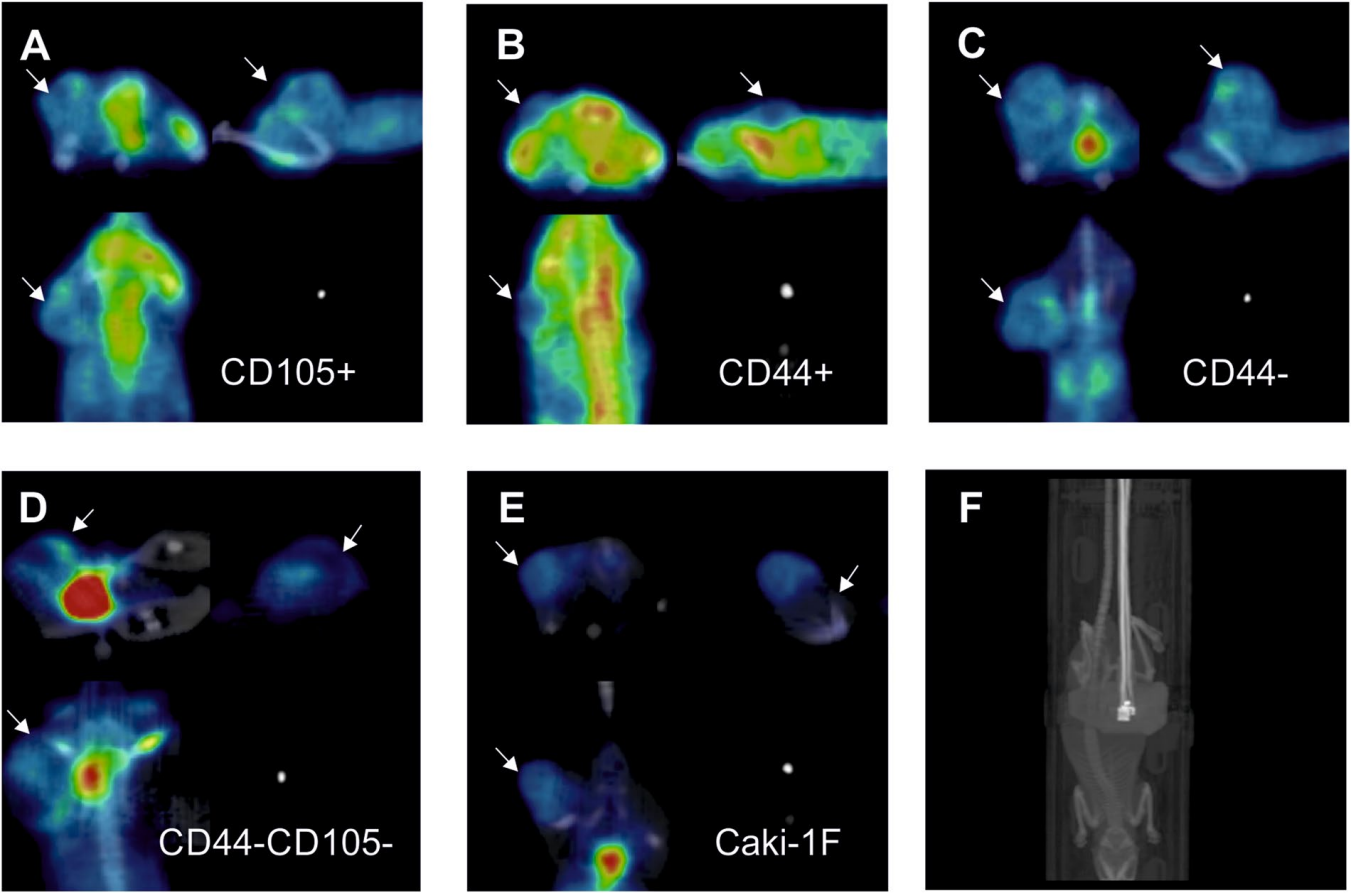
Representative PET images after intravenous administration of 18FDG. Various populations of Caki1F cells: CD105+ (**A**), CD44+ (**B**), CD44−, CD44−/CD105− (**D**) or the unsorted Caki-1F cells (**E**). The last panel (**F**) shows a representative CT scan. Arrows point localization of the tumor.

Tumors induced by unsorted Caki-1 cells did not reveal enhanced uptake of [18F]FDG (Fig. 11E). Similarly, all the tumors induced by sorted subpopulations of Caki-1 cells were not visualized by FDG-PET.

### Immunohistochemistry (IHC) of CD105+/— CSC xenografts

**Figure 12:**
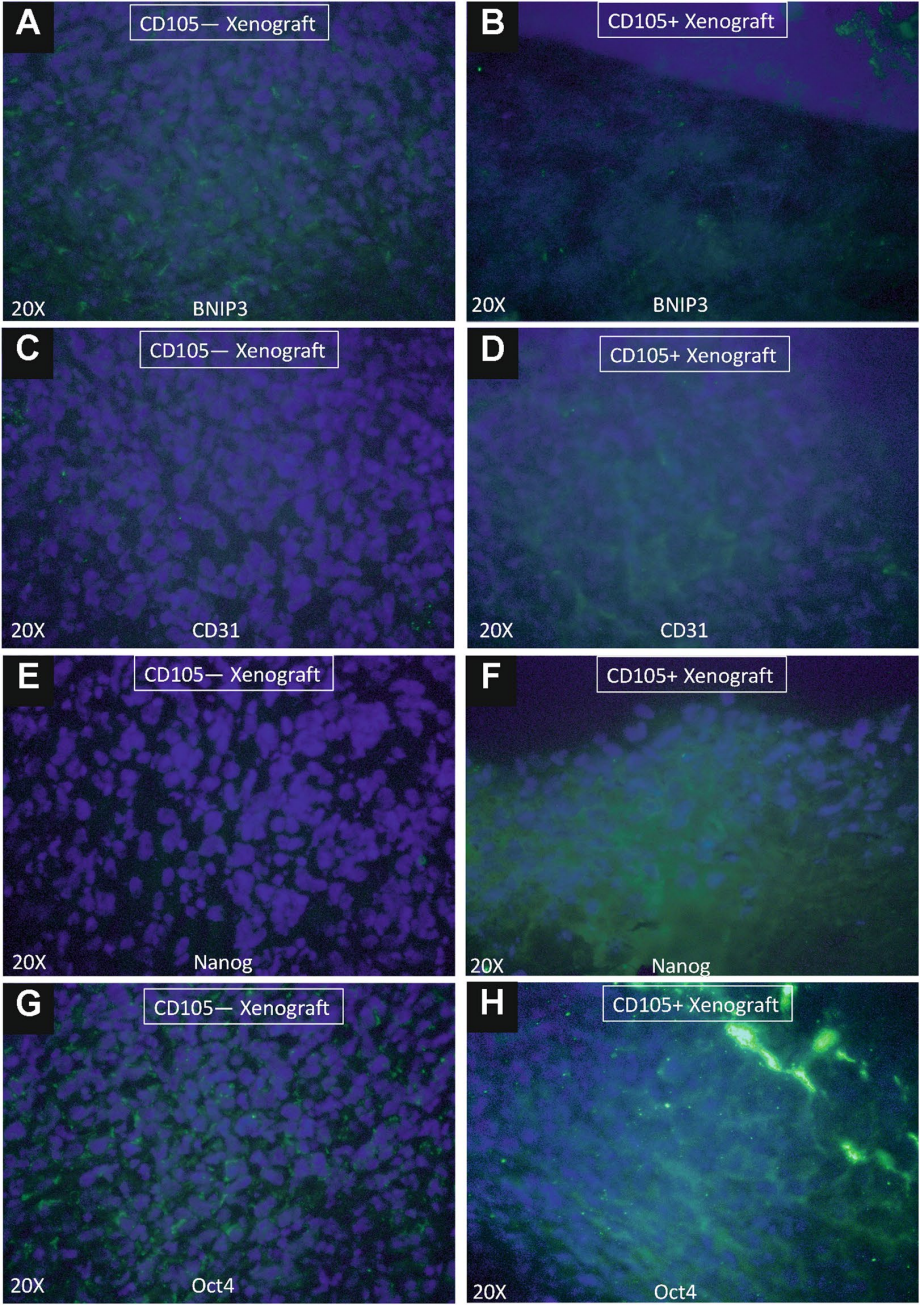
Immunocytochemistry of tumor xenografts. Immunocytochemical staining of BNIP3, CD31, Nanog and Oct4 in tumors derived from CD105−**(A) (C) (E) (G)** and CD105+ **(B) (D) (F) (H)** xenografts. Merged fluorescence of Alexa Fluor 488 (green) and Dapi (blue) is shown in all images.

**Figure 13:**
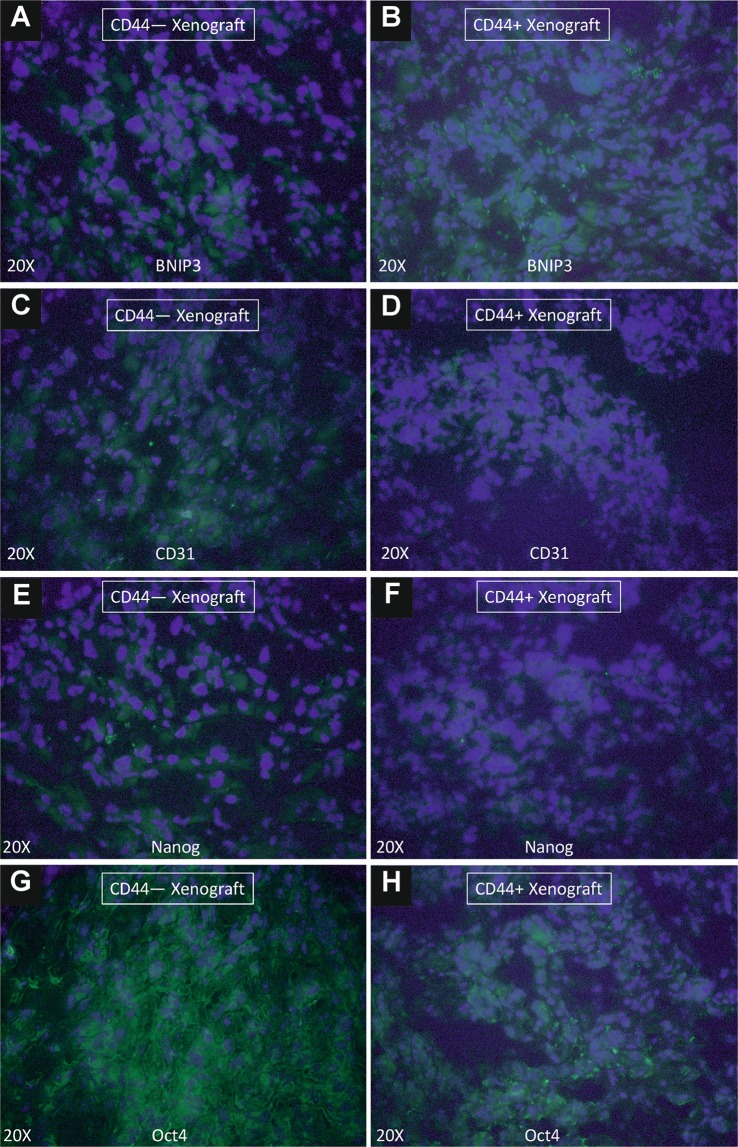
Immunocytochemistry of tumor xenografts. Immunocytochemical staining of BNIP3, CD31, Nanog and Oct4 in tumors derived from CD44−**(A) (C) (E) (G)** and CD44+ **(B) (D) (F) (H)** xenografts. Merged fluorescence of Alexa Fluor 488 (green) and Dapi (blue) is shown in all images.

We have found that CD105− tumor xenografts were enriched in expression of BNIP3 protein when compared with the CD105+ tumor xenografts (Fig. 12). Tumor from CD105− xenografts were found negative for CD31, while CD105+ xenografts were enriched in CD31+ cells. Cells of CD105+ tumors expressed Nanog and Oct-4 proteins, while cells from CD105− tumors only Oct-4 protein (Fig. 12). At the same time CD44+ and CD44− tumor cells did not express BNIP3, while CD44− xenografts were enriched in CD31+ cells. Cells of CD44− xenografts overexpressed Nanog and Oct-4, while cells from CD44+ xenografts show very low expression of these stem-related markers (Fig. 13).

## Discussion

In order to analyze appropriate RCC subtype specific cell lines must be selected based on their confirmed histology^9^. Data on the expression of markers should be interpreted along with the origin of RCC cells including tumor type and site of origin^8,20^. For initial characterization of RCC-CSCs candidates preselected cell surface were used and cells were isolated including CD105+ cells^13^ and CD133+ cells^15^ from nephrectomy specimens (primary tumor); CD44+ (ALDH1+) cells - from 293T human embryonic kidney cell line^10,11^; CXCR4+ cells from RCC-26, RCC-53 cell lines^17^. The RCC-CSCs have also been isolated based on the fundamental stem cell feature of self-protection by dye-exclusion capacity, and they are referred with respect to low fluorescence side populations (SP). These SP cells in RCC were isolated by rhodamine 123^23^ or Hoechst 33342 from primary tumors (nephrectomy specimens) and also ACHN and 769P cell lines^11,24–26^. Cells were also selected based on metabolic biomarker - ALDH1+ cells isolated from ACHN and KRC/Y RCC cell lines^11^ or with and synchrotron radiation-Fourier transform infrared (SR-FTIR) spectroscopy profile^27^. As it was established before tumor formation in immunodeficient mice (i.e. xenograft model) identifies cells with stem cell properties^3^ and in the case of our subpopulations analyzed both CD105+, CD44+, as well as CD105−, CD44− and CD105−/CD44− gave rise to tumor growth in mice. Growth of multiple sublineages of cells confirms that single-marker based characteristics of CSCs is incomplete and that co-expression of markers should be taken in mind when describing CSCs phenotype.

Presence of CD105+ subpopulation has been previously analyzed in 786-O, ACHN, OS-RC-2, CAKI-1, and SN12-PM cell lines and the highest level of expression was described for highly metastatic cells line SN12-PM6 and lowest for primary-tumor derived 786-O cell line^28^. We have shown that CD105+ subpopulation is actually present in most of RCC cell lines (SMKT-R3, Caki-2, 786-O, 769-P, RCC-6, Caki-1, ACHN and that CD105+ cells express also alkaline phosphatase (AP)^12^. The exact size of CD105+ cells subpopulation within the cell lines range from 0.03% (786–0), 0.06% (ACHN) to 2.17% (OS-RC2), but in as much as 90.93% (Caki-1) to 93.9% (SN12PM6) cells may be positive^28^. In RCC the association of CD105 with different disease stages is complex, but CD105 may through translation initiation of targeted genes promote RCC progression^29^. High CD105 mRNA expression was previously associated with RCC metastases and high tumor stage^29^. In RCC cell lines CD105 was reported by us to be highly expressed in metastatic Caki-1 cell line before^12^, as well as by other groups^28^. Moreover, detection of CD105 marker in peripheral blood is considered to be effective tool to evaluate the early metastasis in RCC, especially in a cancer stem cell -positive conditions^30^. CD105 was shown to play functional role in maintaining cancer stem cell phenotype and epithelial – mesenchymal transition (EMT) phenotype^31^, while cells after EMT disseminate to distant organs and form metastases^32^. Due to high expression of the CD105 marker, also in our previous study (4%)^12^ Caki-1 cell line was used in the present study. As CD105−expressing subpopulation in human RCC xenografts from patient (nephrectomy) samples was shown to possess high capability of self-renewal and ability to form spheres in cell culture *in vitro* in complement to tumors in mice^28^ it remains interesting that CD105− cells also give rise to tumors in our study. It is known that TGFβ pathway activation promote RCC 3D sphere formation and CD105 expression is up-regulated in stem-promoting conditions^33^. RCC cells derived spheres have actually been shown to exhibit cancer stem cell (tumor initiating cell) properties: self-renewal, tumorigenicity and the ability to differentiate into other cell types of original tumor^34^. On the contrary, knockdown of CD105 by short hairpin RNA or CRISPR/cas9 reduced stemness markers expression (OCT, NANOG, SOX-2) and sphere-formation ability. CD105 silencing accelerate senescence *in vitro* and significantly decreased tumorigenicity and gemcitabine resistance^28^. Nevertheless, CD105 expression seems to be related with stemness in ccRCC, but not other subtypes of RCC^20,35^ and RCC cell lines based results must be interpreted in relation to their pathology (subtype) and origin^9^.

In healthy individuals CD44 is hardly expressed in the kidney except for passenger leukocytes^36^. Moreover, no CD44 expression is detectable in non-ischemic kidneys. On the contrary, CD44 is expressed in proximal tubular cells after ischemia. CD44 protein localize in basal and lateral kidney cell membranes^37^. Besides ischemia, also in inflammatory kidney diseases, CD44 expression is up-regulated in crescents of Bowman capsule and in injured tubular cells^36^. At the same time CD44 is a marker of parietal epithelial cells in glomeruli with stem-like phenotype of adult kidney. As feature of stemness CD44+ cells are able to differentiate into podocytes and proximal tubular cells^36^. In terms of function CD44 ectodomain contains binding sites for hyaluronic acid (HA) that enables its interaction with growth factors (FGF, EGF, VEGF), growth factor receptors (PDGFR, c-MET, ErbB2) and matrix metalloproteinases (MMP7, MMP9, MT-MMP1). CD44 as a transmembrane adhesion glycoprotein, and hyaluronan receptor, participates in the uptake and degradation of hyaluronan^38^. It is therefore possible that the CD44−null stem-like cells are disadvantaged in multiple ways — with reduced motility and HA and matrix proteins binding^39^. Moreover, interesting functional CD105−/CD44+ interaction has been described. Signaling of TGF-β1 via Smad-2 and Smad-3 phosphorylation and nuclear translocation is actually reduced in CD44^−/−^ kidney cells^36^, so CD44− cells may function actually as CD105− cells. In fact, after binding with HA, CD44 protein interacts with TGF-β receptor I, and enhance TGF-β1 signaling because the matrix metalloproteinase-9 (MMP-9), bounded with this complex, cleave pro-TGF-β1 into its active form^36^. At the same time in RCC (in Caki-1, Caki-2, ACHN, and 786–0) cells CD44 expression was shown to be up-regulated by Twist2 - member of the basic helix-loop-helix (bHLH) family^40^. In breast cancer model it has been shown that HIF-1α as a regulator of CD44 that increased the number of CD44 molecules and the percentage of (variant exons v6 and v7/8) CD44 positive cells is higher in cancer cells in hypoxia^41^, which might also be true for RCC that is known to harbor VHL mutations. Similar signaling pathway cross-signaling was also reported for other RCC-CSC marker. The enhanced self-renewal activity of the CXCR4-positive spheres is induced by up-regulation of HIF2α expression. Such RCC-derived spheres present undifferentiated phenotype *in vivo* and form subcutaneous tumors in mice. In return propagation of RCC cell lines (Caki-1, Caki-2, 786-O, 769-P) in anchorage-independent floating spheres promote propagation of CXCR4 (CD184) expressing cells^42^.

Finally, our latest results stay in accordance with most recent reports on co-expression of multiple markers on RCC-CSCs. Spheres derived from anti-miR-17 transfected Caki1 and ACHN RCC cells showed increase expression of both CD44 and CD24^34^.

It has been recently shown that Caki-1, unlike 786-O and A498, display subpopulation heterogeneity when CD44, CD90, CD105 and CD146 marker expression is analyzed. Co-expression analysis of CD44 and CD105 markers revealed CD44High/CD105High and CD44Low/CD105Low subpopulations. These subpopulations had different frequencies in CD90 and CD146 expressing cells. Most frequent were CD146Low/CD90High/CD44High/CD105High as well as the CD146Low/CD90Low/CD44Low/CD105Low population. In particular in Caki-1 cell line subpopulation of CD44Low cells with co-expression of CD90 are less clonogenic, but this is not true for all CD44Low subpopulations. Furthermore CD146Low/CD90Low/CD44High/CD105High and CD146High/CD90Low/CD44High/CD105High subpopulations further differ based on EpCAMLow and EpCAMHigh expression and presented 46.90% ± 6.89% and 6.42% ± 3.43% of cells respectively. EpCAMLow and EpCAMHigh population was also present in the CD146High/ CD90Low/CD44High/CD105High population (0.76% ± 0.45%). In the final analysis of CD73 and CD29 markers expression of CD90High/CD146Low subpopulation all cells had CD73Low/CD29High phenotype. On the contrary, the CD44Low/CD105Low subpopulation of the CD146Low/CD90Low cells was CD29Low. Last three subpopulations presented also low expression of CD73. On the other hand the all subpopulations of CD146Low/CD90Low and CD146High/CD90Low cells expressed high levels of CD29 and moderate to high levels of CD73. CD146High/CD90Low and CD146Low/CD90Low Caki-1 subpopulations in over 80% expressed high amounts of CD44 and CD105. At the same time clonogenic CD146Low/CD90High subpopulation expressed CD44High/CD105High in below 60% of cells and high number of CD44Low/CD105Low gate^43^, which may be relevant to our CD105−/CD44− cells. As the authors of aforementioned study claim, we also believe that increased proportion of CD105 expressing cells does not identify a substantial increase in clonogenic potential, but it is still important in maintaining clonogenicity. In case of CD44 expression its general high amount in multiple RCC cell lines – including 786-O, A498 and Caki-1- suggests that it is actually a marker of mesenchymal phenotype of RCC cells^43^.

Caki-1 cells were previously shown to form morphologically weakly differentiated relatively homogenous xenograft tumors surrounded by fibrous capsules in athymic nude mice^44^. Injection of 10^4^ cells into NOD/SCID/γ(c)(null) (NSG) mice produced 226.3 ± 78.7 mm^3^ tumors after 6 weeks^34^. In our setting the tumors were bigger, i.e. over 500 mm^3^ after 7 weeks which can be explained by larger number of injected cells (10^6^).

Magnetic resonance *time of flight* angiography revealed formation of new vessels in tumors formed by all the cell subpopulation that appeared to be tumorigenic. This rather qualitative than quantitative approach is widely used for assessment of neovascularization in animal models of malignancies, in particular for in studies that involve anti-angiogenic drugs, e.g. Ziegler, *et al*.^45^. Various MR angiographic approaches has been extensively characterized and validated with histologic studies that proved their reliability^46,47^. Time of flight MR angiography, an approach that we used in this study, despite relatively low spatial resolution (in comparison to CT angiography) provides good contrast to noise ratio (CNR) in preclinical settings^48^. Moreover, *time of flight* MR angiography was proved previously as a useful method for tracking effects of anti-angiogenic treatment in an animal model of RCC^49^.

Relaxometric MRI techniques (T1/T2 mapping) provide quantitative data on magnetic properties of the tissue. In contrast to standard imaging techniques (T1-weighted, T2-weighted, T2*-weighted that are susceptible to various not fully controlled factors besides the ‘real’ relaxation times values) they actually measure the relaxation times and allow creation of relaxation times ‘maps’ (parametric images). T1 shortening in tumors is thought to indicate necrosis because of release of complexed paramagnetic ions from necrotic cells^50^, in particular in response to chemotherapy^51^. On the other hand, T1 elongation may be a result of increased water content in the extracellular space and possibly correlate with elevated tumor interstitial pressure^52^. In tumors that developed after implantation of CD105−/CD44−CD44− cells we have noted T1 elongation in contrast to all other tested subpopulation of cells. It may suggest that the microstructure/microenvironment of the CD105−/CD44− tumors was different than in the tumors induced by injection of other Caki-1 subpopulations.

*In vivo* localized magnetic resonance spectroscopy provide insight into metabolic activity of the tumors. One of the most important signal is the choline signal present at 3.20 ppm in magnetic resonance spectroscopy. It is in fact a sum of signals from trimethylamine groups in three choline compounds: glycerylphosphocholine, phosphocholine and free choline. High choline signal is regarded as a biomarker of elevated lipid membranes turnover and proliferation^53^. Moreover, choline is an established biomarker of malignancy, especially in breast cancer^54^ and proposed as a biomarker of malignancy in RCC^55^. Choline compounds are present in healthy renal tissue^56^. However, in RCCs levels of choline compound measured by MRS *in vivo* is elevated and correlate with aggressiveness of the tumors^57^. High choline signal was present in tumors developed from all the cell subpopulations that generated tumors in our setting, confirming their malignancy. Another confirmation of the high proliferation was high lipid signal, in particular the Lip09 (at 0.9 ppm) and Lip13 peaks (at 1.3 ppm). Presence of lipid droplets was demonstrated in RCC cells^58^ and high MRS lipid signals were shown RCC tumors^55^. Interestingly, we noted presence of detectable taurine signal only in CD44−/CD105− tumors. Taurine levels are low in healthy renal tissue. However, it was found to be elevated in papillary RCC^56^. It may suggest that the CD44−/CD105− tumors resemble some features of papillary RCC.

Currently, RCC is most often detected accidentally when performing other cross-sectional imaging studies^59^. In patients with suspected RCC, the decision-making treatment of patients is the determination of the histology and malignancy of a potential tumor before the implementation of the operating procedure, and determination of the metabolic profile of primary and metastatic changes, if any are present, to predict behavior and outcome in response to metabolic treatment. Usefulness of FDG PET in RCC was discussed in several recent papers both experimental and reviews, e.g.^60–65^. Some of the authors point that [18F]FDG is excreted by kidneys and its use as a routine tracer could be challenging^66–68^. However, it might be useful in detection of metastasis^69^ or for monitoring of treatment response in molecular therapies of RCC^70^. The use of PET could also provide supplementary prognostic information and may help to personalize the therapy. RCC is cancer with spontaneous and various glucose nutrition^71^, therefore additional evaluation using fluorodeoxyglucose in PET/CT tests may prove essential for the second decision point, due to the selection of targeted drugs for specific metabolic cancer profiles. There are reasons to believe that conducting [18F]FDG PET/CT testing is a reasonable addition to the information on the development and therapy of RCC with limited tissue testing. This is crucial to reduce invasive procedures for both the patient and the biological sample is taken, whose path from sampling to the final result is subject to a large analytical error. The biological structure of the sample may also change or be damaged by many analytical procedures. Ozulker *et al*.^72^ examined 18 patients with “suspicious” kidney masses using [18F] FDG PET/CT. They determined a given mass as malignant if the intensity of radiotracer uptake was greater than the intensity of accumulation in the renal parenchyma. Basing on their assignments, a useful conclusion can be drawn that half of the ccRCC tumors are positive for FDG-PET. Another group showed a strong correlation between SUV base and size change on CT of tumors in ccRCC^73^. Majhail, *et al*.^74^ found that a positive PET result confirms the malignancy of the tumor and may be useful in detecting distant metastases. In experimental settings it seems that enhanced [18] FDG uptake may depend on the populations/subpopulation of cells used for induction of the tumor in experimental animals. eg. 786-O xenografts were shown to display increased [18F]FDG uptake (SUVmax>10)^75^ but not xenografts induced by injection of ‘unsorted population’ of Caki-1 cells orSK-RC-52 cells^76^. In our study [18F]FDG uptake was moderate in all tumors induced by injection of all sorted subpopulations of Caki-1 cells in our study and failed to visualize the tumors. This may indicate no remarkable differences in glucose turnover between these tumors.

## Conclusion

We have shown that CD44−CD105− subpopulation of Caki-1 displaying stem-like phenotype. Tumors induced by this subpopulation show specific features distinct from other tumors induced by the other tested subpopulations in terms of relaxometric properties and metabolic fingerprint. For future research in the RCC field co-expression of multiple markers will be crucial to define stem cell signature. Primarily reported CD105+ cells seem to harbor further subpopulation of different tumorigenic potential.

## Methods

### Cell culture

Human metastatic (Caki-1) clear cell RCC cell line was obtained and cultured as previously described^12,77^. Sorted CSCs/TICs (CD105+/−, CD133+/−, CXCR4+/−, and CD44+/−) cells were cultured in FreeStyle 293 Expression Medium (ThermoFisher Scientific, Massachusetts, USA). Confluent cell monolayers were harvested with Accutase Cell Detachment Solution (BD Biosciences, California, USA).

### Flow cytometry analyses and cell sorting

To examine the CD105, CD133, CXCR4 and CD44 cell population within RCC cells, the cells were disassociated with Accutase Cell Detachment Solution (BD Biosciences, California, USA) and prepared as single cell suspensions. Cells were stained separately with fluorescent conjugated CD105, CD133, CXCR4 and CD44 according to the manufacturers’ protocols along with the appropriate unstained controls. For co-expression experiments Caki-1 cells were prepared and labelled in a single tube with CD133 PercP, CD44−PE, CD105−FITC, CXCR4-APC in pairs. FACSCalibur (BD biosciences, California, USA) was used for cell analysis and selected markers expression measurement. FACSAriaII (BD biosciences, California, USA) was used for cell sorting. Ten thousand cells were used for flow cytometry (FACScalibur) analysis. Flow cytometry data analysis, dot plots, and histograms were prepared using FCS Express 5.1 (DeNovo software, California, USA) as described previously^12,77^. We used two human endothelial progenitor cells (HEPC-CB.1 and HEPC-CB.2) and caco-2 cells as a positive control for CD133 and CXCR4 antibody staining **(**Supplementary Figure 1**)**.

FITC anti-human CD105 Antibody (323204, BioLegend, California, USA), PE anti-CD133/2 (130–090–853, Miltenyi Biotec GmbH, Bergisch Gladbach, Germany), APC anti-human CD184 (CXCR4) Antibody (306509, BioLegend, California, USA), PE anti-CD44 antibody (130-095-180, Miltenyi Biotec GmbH, Bergisch Gladbach, Germany) were used.

### Tumor sphere formation and staining with stem-like (CSC) markers

RCC cells were counted and seeded at density of 100 cells/well in ultra-low attachment 24 wells plates (TC plate, suspension, F, Sarstetd, Numbrecht, Germany) supplemented with sphere promoting media as described previously^20,77^. Later culture media was removed, and tumor spheres were rinsed briefly in PBS (3 times). 10% goat serum in PBS was used for blocking at room temperature (1 hr). Tumor spheres were incubated separately with diluted primary antibodies against CD105 (1:1000), CD133 (1:500), CD44 (1:1000) and CXCR4 (1:1000) at 4 °C for 4 h; washed three times with PBS and incubated with Alexa Fluor 488 secondary goat anti-mouse antibody (1:400) for 1 h at room temperature. The spheres were rinsed 3 times with PBS, followed by incubation with DAPI (1:5000; ThermoFisher Scientific, Massachusetts, USA) for 10 min. As control the spheres were incubated only with secondary antibody. The slides were washed with PBS and covered with coverslips using CoverGrip Sealant (Biotium, California, USA), and images were captured using an Olympus CKX41 fluorescence microscope.

### Animals

Male NOD SCID mice (NOD.C.B-17Prkdc(scid)/J, 4–5 weeks) were obtained from Charles River Laboratories (Wilmington, MA, USA). Animals were kept in individually ventilated cages with food and water *ad libitum*. All animal experiments were performed in accordance with the EU Directive 2010/63/EU for animal experiments and respective local regulations. Animal research followed also internationally accepted guidance for the care and use of laboratory animals, including the National Institute of Public Health – National Institute of Hygiene (NIPH – NIH) guidelines, and was approved by the IV Warsaw Local Ethics Committee for Animal Experimentation (National Medicines Institute, 30/34 Chelmska Street, 00–725 Warsaw, PL, permission No. 93/2012 with up-dates No. 46/2015, 47/2015, and 87/2015).

### Implantation of RCC cells

The implantation procedure was performed according to a protocol by Morton and Houghton^78^. The procedure was performed in sterile conditions: the surgery was performed inside biosafety cabinet with laminar flow of sterile air and with the use of sterile surgical tools. Animals were anesthetized with isoflurane (induction 4%, maintenance 1.5–2%; Baxter, Deerfield, IL, USA) in oxygen and the place of injection (left dorsal flank) was shaved and wiped with 70% ethanol solution.

Cells were suspended in PBS and Matrigel mixture (1:1, v:v; Sigma-Aldrich, St. Louis, MO, USA) immediately before the surgery and kept on ice until the implantation^79^. 200 µl of such suspension containing 10^6^ cells was injected into subcutaneous space through 21G needle^34,80^.

### MRI

The measurements were performed with 7T Bruker Biospec tomograph (70/30 USR, Bruker Biospin, Ettlingen, Germany). Animals were anesthetized with isoflurane (induction 4%, maintenance 1.5–2%). Birdcage transmit-receive cylindrical radiofrequency volume coil (40 mm inner diameter) was used. Respiration rate and body temperature were monitored during the experiment with small animal monitoring system (SA Instruments, Stony Brook, NY, USA).

The imaging protocol included:

1. anatomical T2-weighted imaging of the tumors in the axial plane (TurboRARE sequence, TR/TE_eff_ = 2600/30 ms, RARE factor = 4, NA = 4, FOV = 30 mm × 30 mm, resolution 117 µm × 117 µm × 500 µm, 30 slices with 0.1 mm gap);
2. anatomical T2-weighted imaging of the tumors in the coronal plane (TurboRARE sequence, TR/TE_eff_ = 2200/30 ms, RARE factor = 4, NA = 5, FOV = 30 mm × 30 mm, resolution 117 µm × 117 µm × 600 µm, 25 slices with 0.1 mm gap);
3. MR angiography (FLASH *time of flight*, TR/TE = 17/4.5 ms, NA = 4, FOV = 30 mm × 30 mm, resolution 117 µm × 117 µm × 500 µm, 60 slices – overlapping with 0.2 mm overlap);
4. T1 parametric imaging (measurement of T1 relaxation times; 2D Saturation Recovery Spin Echo Sequence with varying repetition times TRs = 245.3 … 5000 ms; TEeff = 22 ms, RARE factor = 4, NA = 2, FOV = 20 mm × 20 mm, spatial resolution = 234 µm × 234 µm × 1000 µm, 4 slices with 0.2 mm gap).
5. T2 parametric imaging (Multi-Slice Multi-Echo with varying echo times TEeffs = 13 … 416 ms, TR = 5000 ms, NA = 1, FOV = 30 mm × 30 mm, spatial resolution = 234 µm × 234 µm × 1000 µm, 4 slices with 0.2 mm gap).

Anatomical images were manually segmented with OsiriX software (version 5.8.2, osirix-viewer.com) by an operator unaware of the animal groups. Relaxation times were calculated for ROIs covering the tumors with the ISA module of Paravision 5.1 software (Bruker, Ettlingen, Germany).

### Magnetic resonance spectroscopy

The spectra were acquired with the PRESS sequence (TR/TE = 2000/20 ms, 512 averages, 2048 points, scan Time = 17 min) with VAPOR water suppression. Single volume of interest (VOI, 3 mm × 3 mm × 3 mm) was selected for each tumour (Fig. 12F). The acquisition of MR spectra was preceded by an extensive shimming procedure including linear and second order global shims, followed by local shimming with the FASTMAP protocol within the VOI. Spectra were analyzed with the LCModel software^81^.

### PET/CT

PET and CT scans were conducted using Albira PET/SPECT/CT Preclinical Imaging System (Bruker, Billerica, Massachusetts). Animals were anesthetized with isoflurane (induction 4%, maintenance 1.5–2%). 8–10 MBq of [18F]FDG (18F-Fludeoxyglucose) in 100–150 µL was injected intravenously. [18F]FDG was a commercial product Gluscan 500 (Advanced Accelerator Applications Sp. z o. o., Warsaw, Poland). Through the course of radiopharmaceutical uptake and image acquisition respiration was monitored. Scans were started 60 min after [18F]FDG injection. Emission data were collected for 1, 5, 10 min. Spatial resolution of PET images was 1.5 mm. The CT scan parameters ware set as follow: tube voltage was 45 kVp, tube current was 400 µA, and number of projections was 250. Minimal resolution of CT was 90 µm. PET and CT scans were fused using PMOD software, version 3.307, module Fusion Tool (PMOD Technologies LLC, Zurich, Switzerland). Tumor shape on fused image was contoured on all slices consisting part of tumor. Obtained VOIs (volume of interest) were analyzed quantitatively, and SUV (standard uptake value) was calculated.

### Immunohistochemistry

IHC staining of xenograft tumor specimens were performed as described previously^77^. Anti-BNIP3 antibody (ab10433), anti-CD31 antibody (ab24590), anti-Nanog antibody (ab21624) and anti-Oct4 antibody (ab19857) were used (Abcam, Cambridge, United Kingdom).

### Statistical analysis

Statistical analysis and figures were prepared using GraphPad Prism 8 Software. Kruskal-Wallis test was followed by U Mann-Whitney *post hoc* test. Differences were regarded significant for P<0.05. Data are presented as means ± standard deviation.

## Supplementary information

Supplementary information

## Data Availability

All data generated or analyzed during this study are included in this published article (and its Supplementary Information files).
