# Supplementary material for: Renal carcinoma CD105−/CD44− cells display stem-like properties in vitro and form aggressive tumors in vivo
Source: Sci Rep. 2020 Mar 25;10:5379. doi: 10.1038/s41598-020-62205-6 (PMC7096525; doi:10.1038/s41598-020-62205-6)
Supplement: Supplementary file 1 — Supplementary information [file 41598_2020_62205_MOESM1_ESM.pdf]

Supplementary material

Renal carcinoma CD105-/CD44- cells display stem-like properties *in vitro* and form aggressive tumors *in vivo*

Fiedorowicz M.[1,*,#], Khan M.I.[2,3,#], Strzemecki D.[1], Orzeł J.[1,4], Wełniak-Kamińska M.[1], Sobiborowicz A[5,6], Wieteska M.[1,4], Rogulski Z.[7], Cheda L.[7], Wargocka-Matuszewska W.[7], Kilian K.[8], Szczylik C.[2,9,10], Czarnecka A.M.[1,2,11]

[1] Mossakowski Medical Research Centre, Polish Academy of Sciences, Warsaw, Poland

[2] Department of Oncology with Laboratory of Molecular Oncology, Military Institute of Medicine, Warsaw, Poland

[3] Current address: Department of Otolaryngology - Head & Neck Surgery, Western University, London, ON, N6A 3K7, Canada.

[4] Faculty of Electronics and Information Technology, Warsaw University of Technology, Warsaw, Poland

[5] Faculty of Medicine, Medical University of Warsaw, Warsaw, Poland

[6] Department of Soft Tissue/Bone Sarcoma and Melanoma, Maria Sklodowska-Curie Memorial Cancer Center and Institute of Oncology, Warsaw, Poland

[7] Faculty of Chemistry, Biological and Chemical Research Centre, University of Warsaw, Warsaw, Poland

[8] Heavy Ion Laboratory, Faculty of Physics, University of Warsaw, Warsaw, Poland

[9] Current address: Department of Oncology, European Health Centre, Otwock, Poland.

[10] Current address: Medical Center for Postgraduate Education, Warsaw, Poland.

[11] Current address: Department of Soft Tissue/Bone Sarcoma and Melanoma, Maria Sklodowska-Curie Memorial Cancer Center and Institute of Oncology, Warsaw, Poland

*Correspondence: mfiedorowicz@imdik.pan.pl

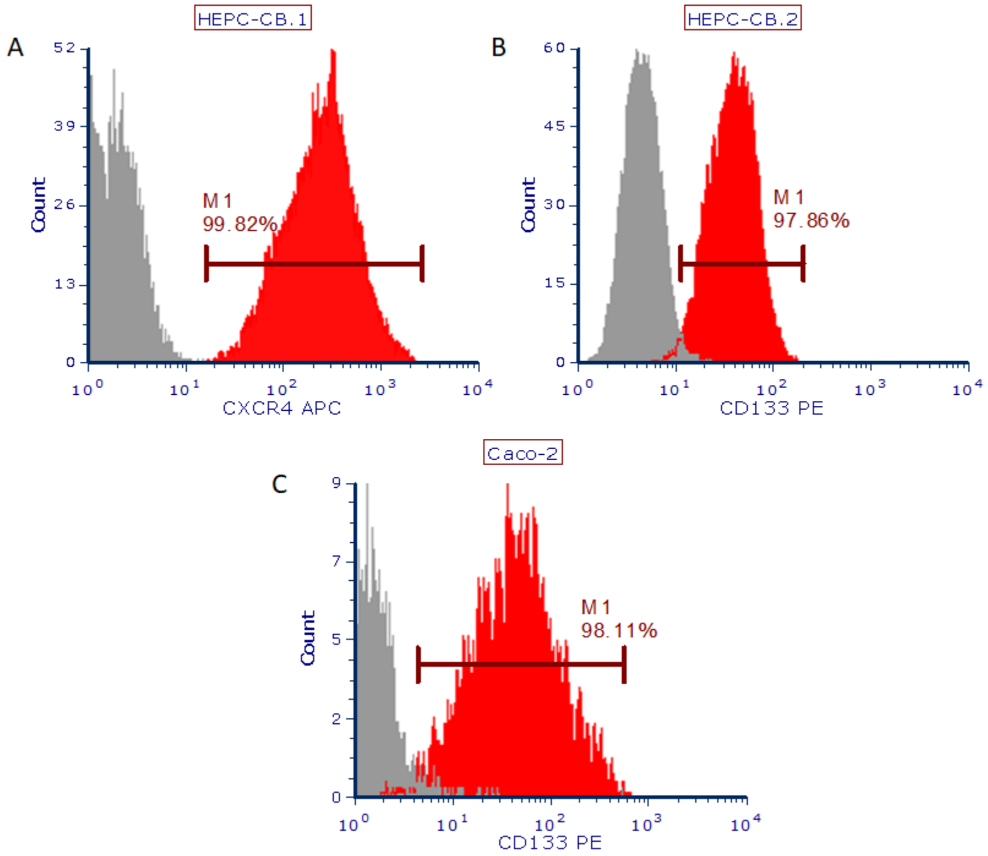

Supplementary Figure 1 – Control cell lines used for expression of CD133 and CXCR4 surface markers. Red histograms showing expression of **(A)** CXCR4 in HEPC-CB.1 cells, **(B)** CD133 in HEP-CB.2 cells, and **(C)** CD133 in Caco-2 cells. Grey histograms represent unstained cells.

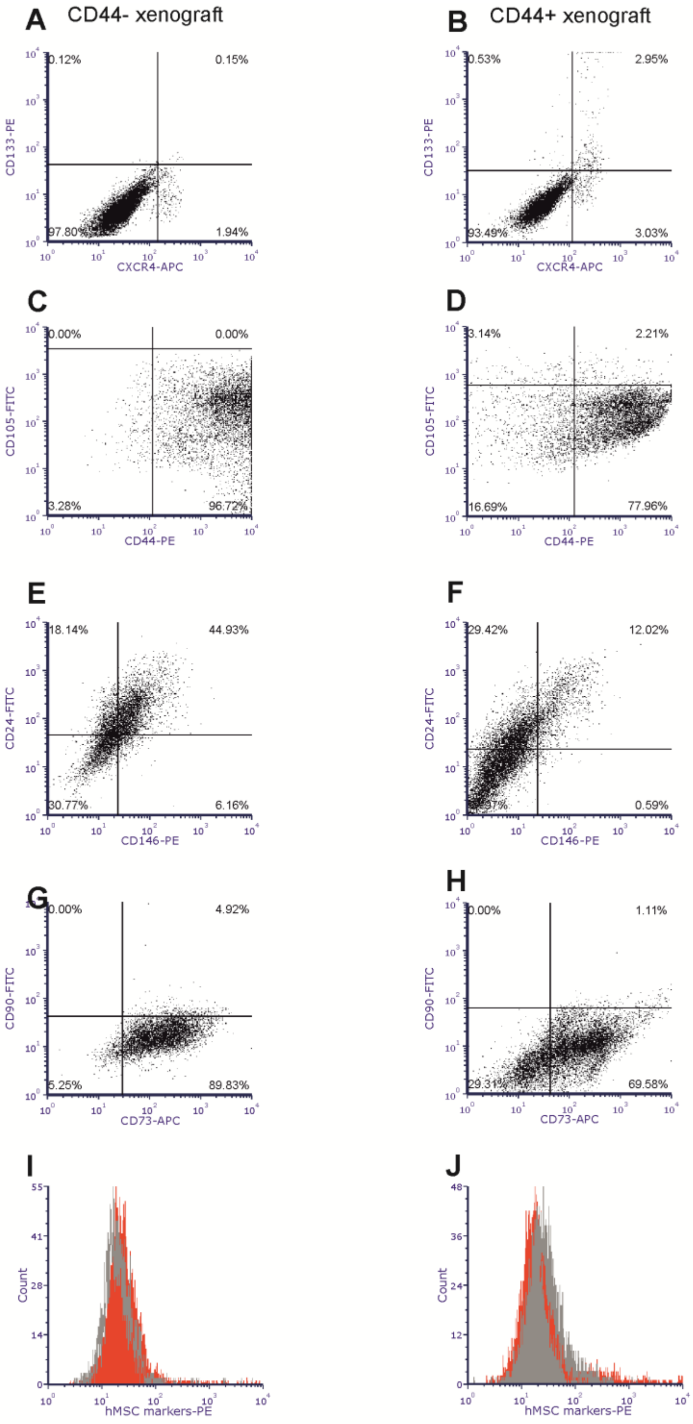

Supplementary Figure 2 – Dot plots showing re-analysis of cancer stem cells (CSC) and human mesenchymal stem cells (hMSC) markers in CD44+/- xenografts. Figure **(A)** and **(B)** showing dual expression analysis of CSCs markers (CD133 and CXCR4) in CD44- and CD44- xenografts, respectively. Figure **(C)** and **(D)** showing dual expression analysis of CSCs markers (CD105 and CD44) in CD44- and CD44- xenografts, respectively. Figure **(E)** and **(F)** showing dual expression analysis of hMSCs markers (CD24 and CD146) in CD44- and CD44- xenografts, respectively. Figure **(G)** and **(H)** showing dual expression analysis of hMSCs markers (CD90 and CD73) in CD44- and CD44- xenografts, respectively. Red histograms **(I)** and **(J)** showing analysis of hMSCs markers (CD34, CD45, CD11b or CD14, CD19 or CD79α) in CD44- and CD44- xenografts, respectively. Grey histograms represent unstained cells.

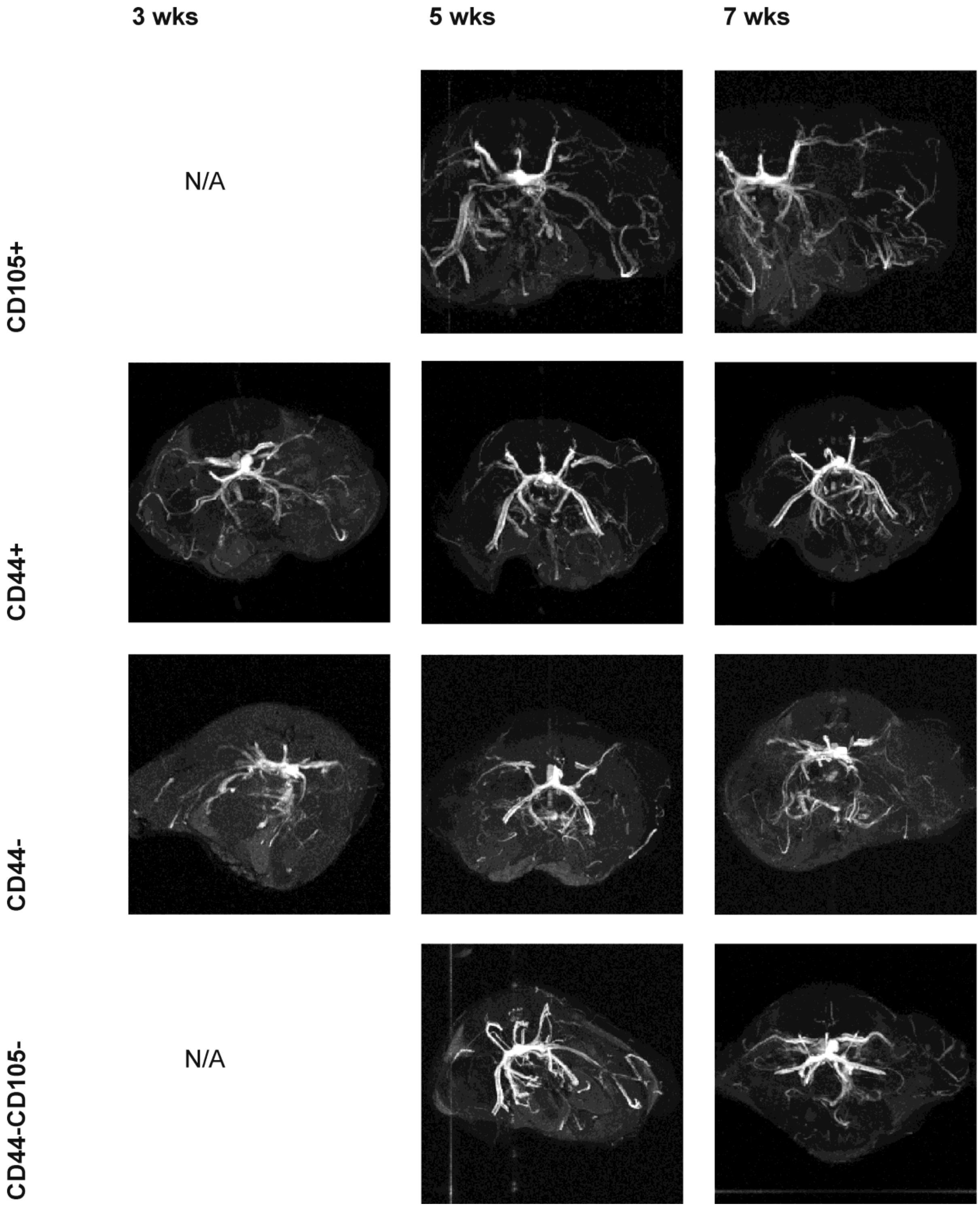

Supplementary Figure 3 - MR *time of flight* angiography in tumors at different timepoints after implantations of various subpopulations of Caki1F cells (3, 5 and 7 weeks after implantation). N/A – tumors not detected in most of the animals, no representative MR angiography.
